# Development of a Virtual Reality Exposure Tool as Psychological Preparation for Elective Pediatric Day Care Surgery: Methodological Approach for a Randomized Controlled Trial

**DOI:** 10.2196/resprot.7617

**Published:** 2017-09-11

**Authors:** Robin Eijlers, Jeroen S Legerstee, Bram Dierckx, Lonneke M Staals, Johan Berghmans, Marc P van der Schroeff, Rene MH Wijnen, Elisabeth MWJ Utens

**Affiliations:** ^1^ Department of Child and Adolescent Psychiatry/Psychology Erasmus University Medical Center (Erasmus MC) Sophia Children’s Hospital Rotterdam Netherlands; ^2^ Department of Anesthesiology Erasmus University Medical Center (Erasmus MC) Sophia Children’s Hospital Rotterdam Netherlands; ^3^ Department of Anesthesia ZNA Middelheim Queen Paola Children’s Hospital Antwerp Belgium; ^4^ Dutch Craniofacial Center Erasmus University Medical Center (Erasmus MC) Rotterdam Netherlands; ^5^ Department of Otorhinolaryngology and Head and Neck Surgery Erasmus University Medical Center (Erasmus MC) Sophia Children’s Hospital Rotterdam Netherlands; ^6^ Intensive Care and Department of Pediatric Surgery Erasmus University Medical Center (Erasmus MC) Sophia Children’s Hospital Rotterdam Netherlands; ^7^ Research Institute of Child Development and Education University of Amsterdam Amsterdam Netherlands; ^8^ Academic Center for Child Psychiatry De Bascule/Department of Child and Adolescent Psychiatry Academic Medical Center Amsterdam Netherlands

**Keywords:** virtual reality, pediatric, anxiety, surgery, anesthesia, intervention, exposure, randomized controlled trial

## Abstract

**Background:**

Preoperative anxiety in children is highly prevalent and is associated with adverse outcomes. Existing psychosocial interventions to reduce preoperative anxiety are often aimed at distraction and are of limited efficacy. Gradual exposure is a far more effective way to reduce anxiety. Virtual reality (VR) provides a unique opportunity to gradually expose children to all aspects of the operating theater.

**Objective:**

The aims of our study are (1) to develop a virtual reality exposure (VRE) tool to prepare children psychologically for surgery; and (2) to examine the efficacy of the VRE tool in a randomized controlled trial (RCT), in which VRE will be compared to care as usual (CAU).

**Methods:**

The VRE tool is highly realistic and resembles the operating room environment accurately. With this tool, children will not only be able to explore the operating room environment, but also get accustomed to general anesthesia procedures. The PREoperative Virtual reality Intervention to Enhance Wellbeing (PREVIEW) study will be conducted. In this single-blinded RCT, 200 consecutive patients (aged 4 to 12 years) undergoing elective day care surgery for dental, oral, or ear-nose-throat problems, will be randomly allocated to the preoperative VRE intervention or CAU. The primary outcome is change in child state anxiety level between baseline and induction of anesthesia. Secondary outcome measures include child’s postoperative anxiety, emergence delirium, postoperative pain, use of analgesics, health care use, and pre- and postoperative parental anxiety.

**Results:**

The VRE tool has been developed. Participant recruitment began March 2017 and is expected to be completed by September 2018.

**Conclusions:**

To our knowledge, this is the first RCT evaluating the effect of a VRE tool to prepare children for surgery. The VRE intervention is expected to significantly diminish preoperative anxiety, postoperative pain, and the use of postoperative analgesics in pediatric patients. The tool could create a less stressful experience for both children and their parents, in line with the modern emphasis on patient- and family-centered care.

**Trial Registration:**

Netherlands Trial Registry: NTR6116; http://www.trialregister.nl/trialreg/admin/rctview.asp?TC=6116 (Archived by WebCite at http://www.webcitation.org/6ryke7aep)

## Introduction

Fifty to 70% of children experience elevated levels of anxiety and distress prior to surgery [1,2]. Preoperatively, anxious children are more often agitated, sad, emotional, less cooperative, and more resistant compared to children who are not anxious [3,4]. Preoperative anxiety is also associated with a higher incidence of emergence delirium, more intense and prolonged pain postoperatively, and poorer recovery [3,5,6]. A child’s operation is also a stressful experience for parents and parental fear has been shown to intensify children’s preoperative anxiety [7,8]. Anxious children undergoing surgery, as well as their parents, are even at risk for a posttraumatic stress disorder [9]. These adverse outcomes underscore the urgent need to develop effective strategies to minimize preoperative anxiety in children.

Education programs have proven to be effective in reducing children’s preoperative anxiety. Nonetheless, a recent systematic review by Copanitsanou and Valkeapää indicated that education seems to have a negative effect on younger children’s anxiety [10]. Other complementary methods for reducing preoperative anxiety in children predominantly focus on distraction, for instance, watching a video, listening to music, playing video games, or distraction by clowns [11-14]. However, scientific literature shows that gradual exposure is a much more effective way to reduce anxiety in children than mere distraction [15]. Due to busy clinical practices and the daily use of surgery rooms, exposing children to all pre- and postoperative aspects is often not feasible.

Virtual reality exposure (VRE) offers the possibility to expose children of different ages to a highly realistic virtual environment that mimics the operating theater of a hospital. Children can get accustomed not only to the operating environment, but also to the procedures associated with anesthesia. VRE has already been shown to be effective as a treatment for specific phobias in children, such as a fear of heights or a fear of flying [16]. To the best of our knowledge, the efficacy of VRE to prepare children for general anesthesia and surgery has not yet been studied. Furthermore, Cochrane reviews showed that most studies examining interventions for induction of anesthesia in children are small and of poor quality [17,18]. As such, high-quality randomized controlled trials (RCTs) are needed.

The aims of the PREoperative Virtual reality Intervention toEnhance Wellbeing (PREVIEW) study are (1) to develop a VRE tool to prepare children for surgery; and (2) to conduct an RCT to test the effectiveness of the VRE tool in children undergoing elective day care surgery.

## Methods

### Virtual Reality Design

The VRE tool encompasses a highly realistic virtual environment that replicates the operating theater of the Erasmus MC-Sophia Children’s Hospital, Rotterdam, the Netherlands.

A multidisciplinary team, consisting of child life specialists, child psychologists, a child psychiatrist, anesthesiologists, a three-dimensional (3D) acting director, and a 3D project manager designed the script of the VRE. Working together with specialized virtual reality (VR) developers and animators, multiple 3D characters, asset, and environment artists created the scenery and character modeling.

During the design and development phases, team meetings were held to review the process and make any necessary adjustments. Overall, the main goal was to create a dynamic and interactive environment that will prepare children for surgery under general anesthesia, in a realistic and child-friendly manner. Once the VR software was created, it was pilot tested in healthy children (N=10). Based on the observations and responses of the pilot test, final adjustments were made.

### Technical Specifications

#### Hardware Details

All 3D characters in the virtual environment were modeled after pediatric anesthesiology employees who had undergone motion capture recording with professional Vicon Motion Systems Ltd equipment (Vicon Motion Systems Ltd). Vicon Vantage cameras were used for body motion capture and the Vicon Cara system, including 4 high resolution high speed cameras and a custom head rig, were used for facial motion capture.

The virtual environment is presented via an HTC Vive headset, using room-scale technology, which allows the user to navigate naturally. Real world awareness is created through a 110 degrees field of view for captivating immersion. The Vive features 32 sensors for 360 degrees motion tracking, a 2160 by 1200 combined resolution, and a 90 Hz refresh rate. With 2 wireless, motion-tracked handheld Vive controllers, each containing 24 sensors, users can interact with precision and experience immersive environments. The headset is connected to a custom computer with an Intel Core i7-5820K processor and a NVIDIA GeForce GTX 1080 graphics card.

#### Software Details

All software used is of professional quality and well known in the film, game, and VR industry. Vicon Blade II software, which is compatible with the Vicon camera systems, is used for body motion capture, whereas Vicon Cara Live and Vicon Cara Post are used for facial motion capture. Blade II provides real-time visualization, in which multiple range of motion sequences, and thus multiple people, can be captured simultaneously. Cara Live is used during setup and capture, while Cara Post automatically identifies and tracks the markers, applied on a human face, over time, creating a 3D point representation of the markers.

With RealityCapture, accurate and realistic 3D models are created out of photographs. 3ds Max and Maya 2016 are digital tools used for creating complex 3D animations and models. Autodesk MotionBuilder 2016 Service Pack 1 is also used for 3D character animation. Mudbox 2016 and Zbrush 4R7 are tools used for high resolution 3D sculpting. Everything comes together in the game engine Unreal Engine 4.13.

### Virtual Reality Storyline

The duration of the VRE intervention is approximately 15 minutes. We developed one version for 4- to 7-year-old children and one for 8- to 12-year-old children such that the explanations of the procedures can be attuned to the child’s developmental level. We used 8 as the cut-off point between the versions because this age represents a key period in children's brain development with respect to cognitive flexibility and information processing [19].

**Figure 1:**
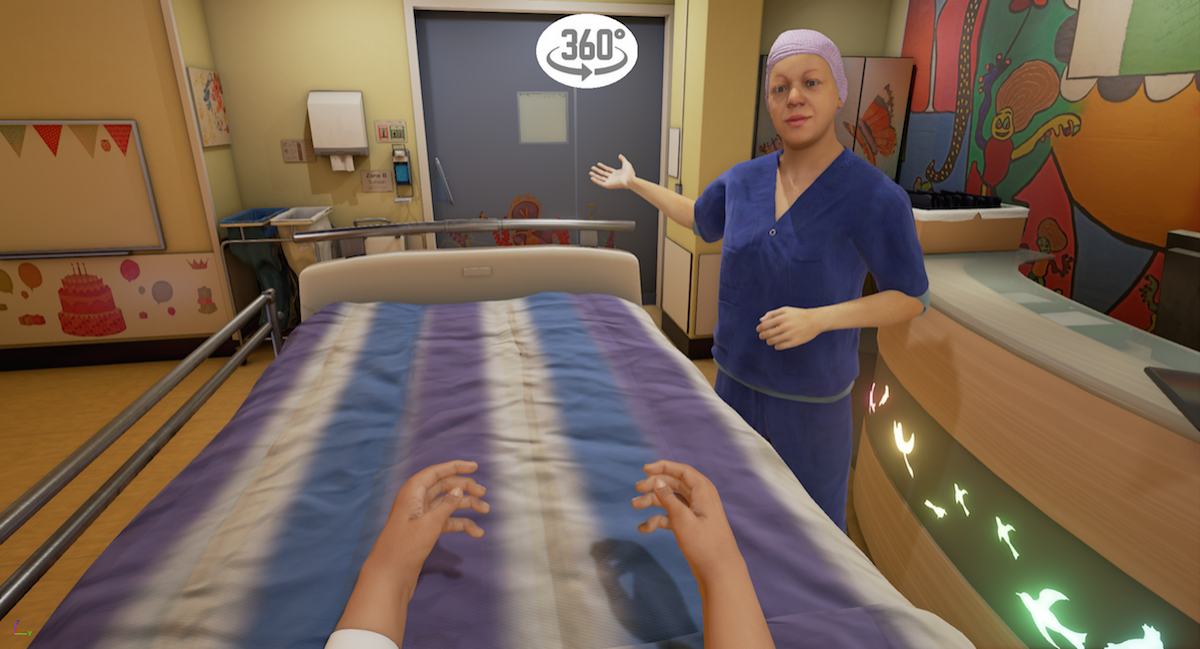
The virtual reality holding area.

**Figure 2:**
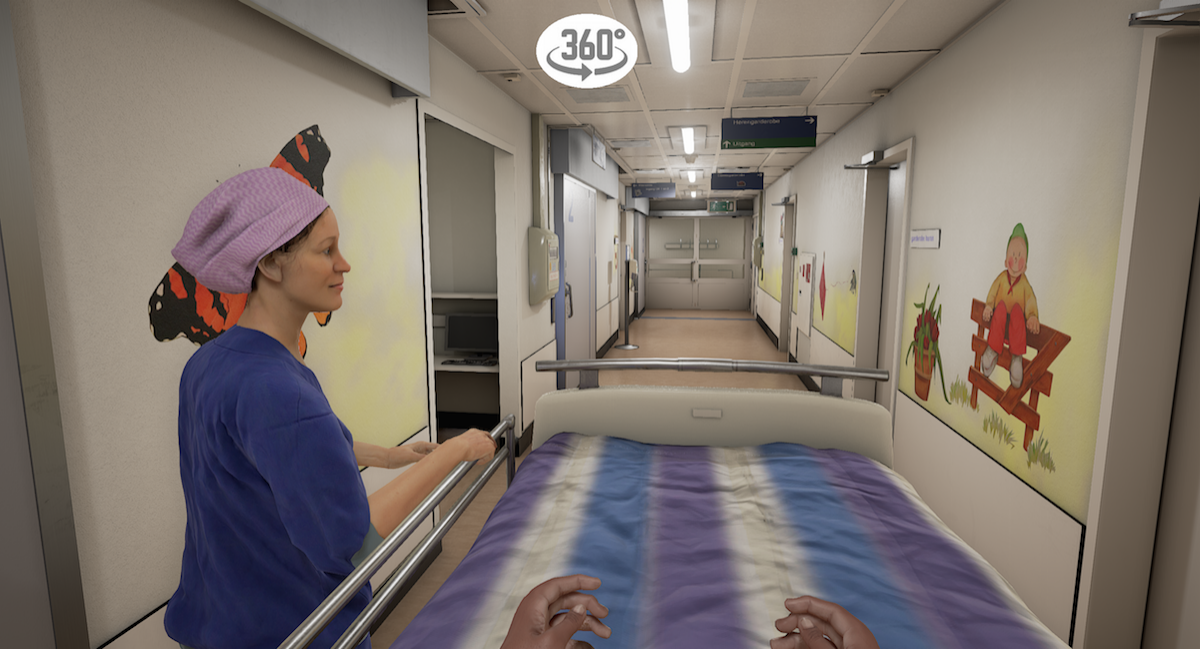
Transportation through the corridor of the operating theater in virtual reality.

**Figure 3:**
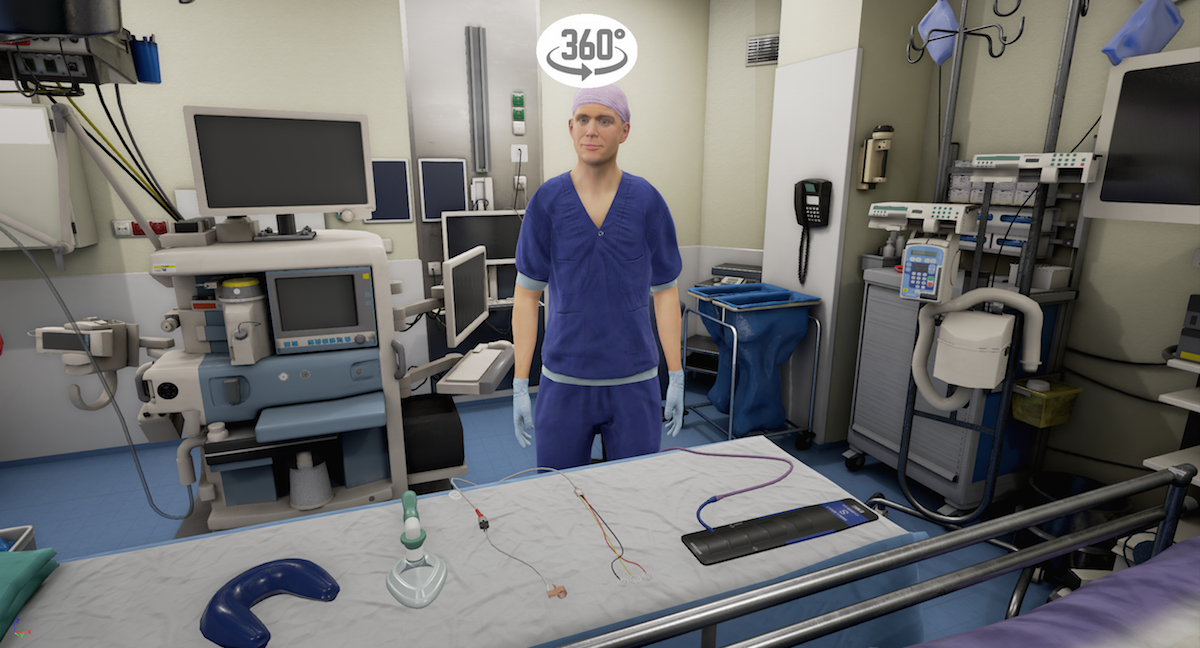
The virtual reality operating room.

**Figure 4:**
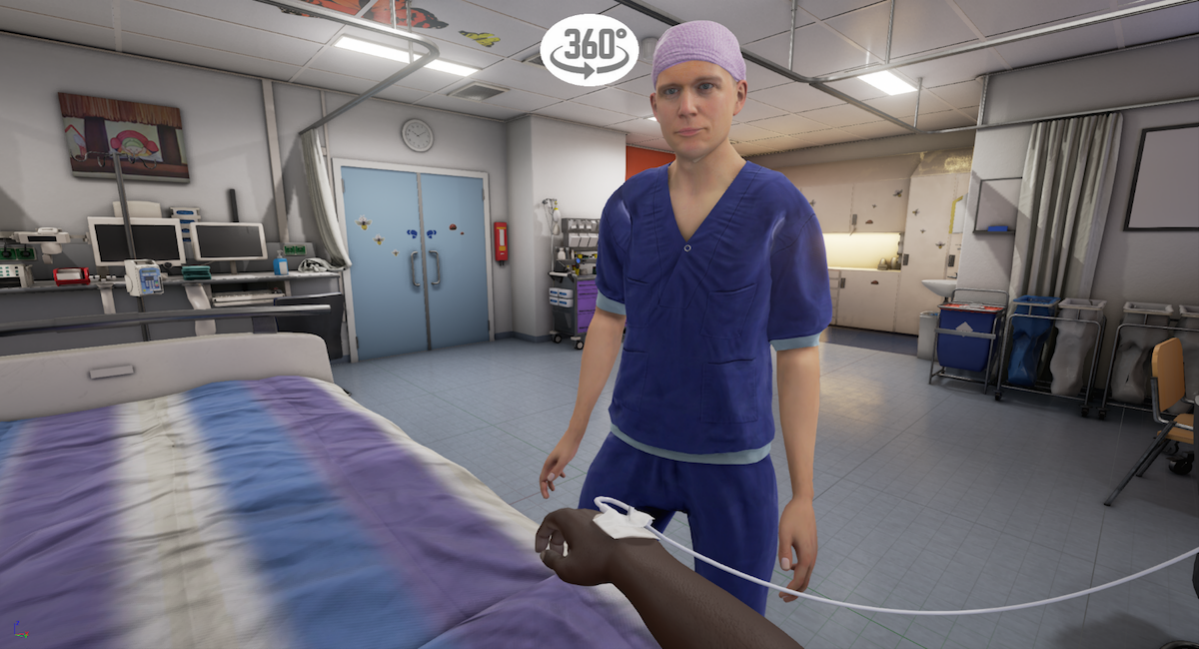
The recovery room in virtual reality.

The VR storyline begins in the holding area, where the child is sitting on a hospital bed (Figure 1). A receptionist welcomes the child and shows him/her a video with extra information on a virtual tablet. The video explains that one of the child’s parents will stay with him/her all the time and shows the hospital gowns the child and parent will be wearing to surgery. After the video, the child in the hospital bed is transported into the corridor of the operating theater by an anesthesiologist and a nurse anesthetist (Figure 2). The bed is taken into the operating room via the surgery preparation room. The child can point at different instruments, such as the oxygen saturation monitor, blood pressure cuff, and anesthesia mask, with a motion tracked controller so that the nurse anesthetist can explain what they are used for (Figure 3). Next, the child moves itself onto the surgery bed where the anesthetic preparation takes place. These preparations are explained at this stage. The program is able to show both intravenous and inhalational induction. After induction, the operating room fades out and the recovery room fades in (Figure 4). Finally, the anesthetist nurse shows another video on a virtual tablet, which explains what kind of feelings the child might experience after surgery, such as pain or nausea.

### Study Design

The PREVIEW study is a single-center, single-blinded RCT carried out in the Erasmus MC-Sophia Children’s Hospital, by the departments of Child and Adolescent Psychiatry/Psychology, Pediatric Anesthesiology, and Maxillofacial, Dental, and Ear-Nose-Throat (ENT) Surgery. This RCT involves a psychosocial intervention (VRE preparation) versus care as usual (CAU) in 4- to 12-year-old children undergoing elective day care dental, oral, or ENT surgery (N=200). CAU involves children being recommended by their anesthesiologist during the preoperative screening consultation to watch the informative online movie of the Erasmus MC-Sophia Children’s Hospital about general anesthesia prior to surgery.

### Inclusion and Exclusion Criteria

Eligible participants are all consecutive pediatric patients (aged 4 to 12 years) undergoing elective day care surgery (ie, dental, oral, or ENT surgery) at the Erasmus MC-Sophia Children’s Hospital, between March 2017 and August 2018. Exclusion criteria are mental retardation, inability of parents to read or write Dutch, epilepsy, visual impairment, or poor general health, indicated by an American Society of Anesthesiologists (ASA) classification of IV or more.

### Patient Recruitment and Procedure

Eligible patients and their parents will be informed about the study by phone and, if interested, receive the patient information folder (PIF) by email. Participation will be voluntary and all data will be anonymized. Both parents will be asked to provide written informed consent. Patients who are 12 years old will also be asked to provide written informed consent themselves. Children under 12 years old will give their permission orally.

After informed consent is provided, children will be randomly allocated to the VRE intervention (N=100) or CAU (N=100) group at hospital admission. Randomization will be stratified by age group (4- to 7- or 8- to 12-years-old), and type of surgery (ie, oral and maxillofacial surgeries, tonsil and adenoidectomy, tympanostomy tubes, or other ear surgeries). The researchers and operating staff will be blinded to group allocation. The research assistant will not be blinded, since he/she will be guiding the intervention. This will take place in a separate room, in the presence of an accompanying parent. Un-blinding takes place if patients are excluded from the study and after the final assessment of the last included patient.

Assessments will be carried out at the following time points: (1) T1, admission to the hospital, before possible intervention; (2) T2, after the VRE intervention or after CAU, in the holding area; (3) T3, during induction of anesthesia, in the operating room; (4) T4, postoperatively, in the recovery room; and (5) T5, 3 days after surgery, at home.

### Sample Size

To conduct a repeated measures analysis of variance (ANOVA) with 4 time points (ie, T1, T2, T4, and T5) for self-reported child anxiety, Cohen *f* of 0.25, an alpha of .05 (2-tailed), and a power of .85, a sample size of 200 patients is needed (100 patients per group). To perform regression analyses with 6 predictor variables, a small to medium effect size, and a power of .85, a sample size of 100 patients in the intervention group is sufficient.

### Outcome Measures

**Figure 5:**
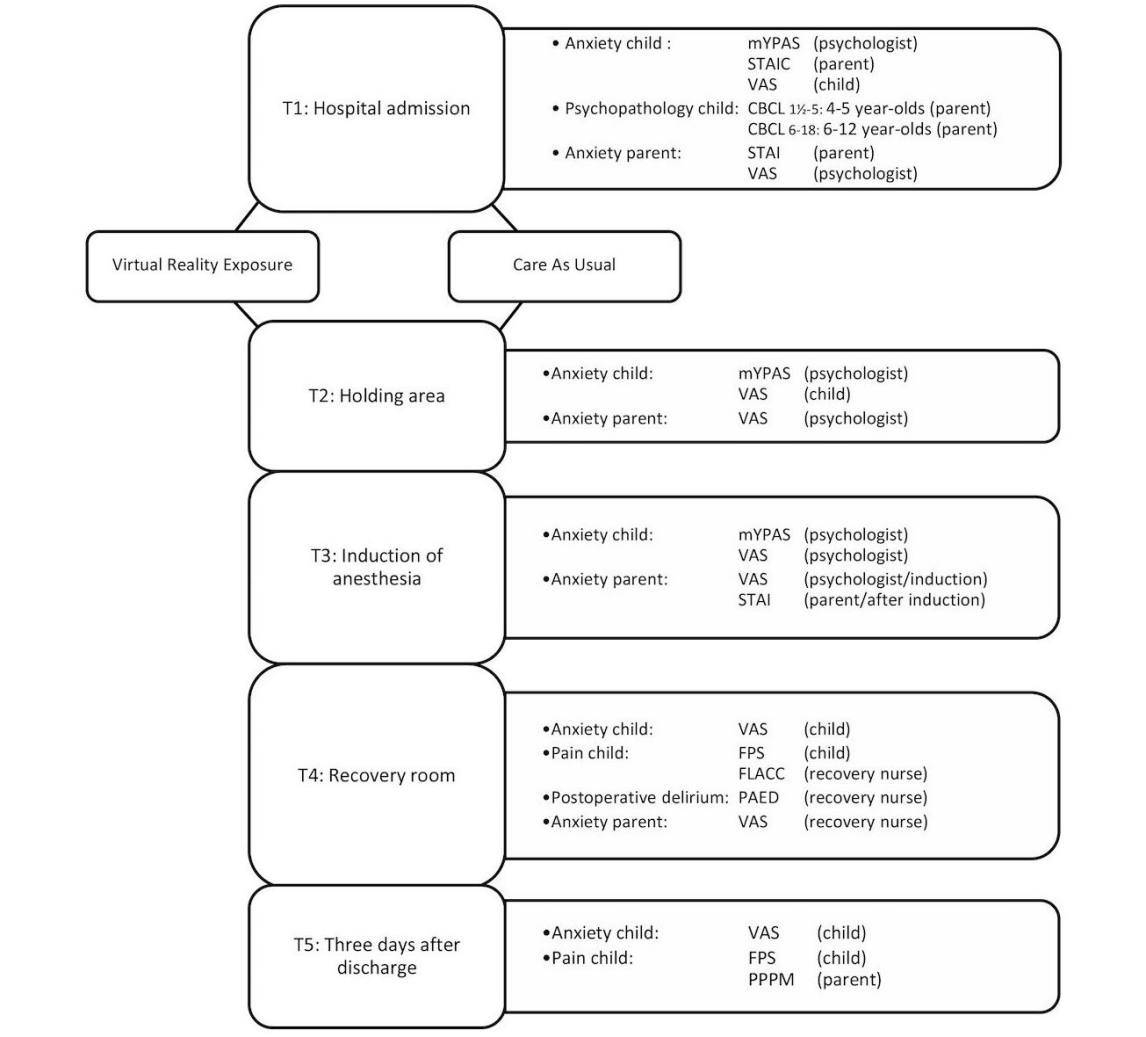
Flow chart of the study design with outcomes, instruments, and informants at each time point.

An overview of the study design and variables at each time point are provided in Figure 5. The primary outcome is change in child state anxiety level between baseline (T1) and induction of anesthesia (T3), evaluated by a psychologist trained in the administration of the modified Yale Preoperative Anxiety Scale (mYPAS) [20,21]. The mYPAS is a commonly used observational tool consisting of 27 items divided into 5 domains: activity, emotional expressivity, state of arousal, vocalization, and use of parents.

Multiple secondary state anxiety outcomes will be examined. Children will indicate their level of anxiety with a Visual Analogue Scale (VAS) at different time points (T1, T2, T4, and T5) [22]. Moreover, situational parental anxiety, both pre- and postoperatively will be self-reported using the state anxiety form (20 items) of the State-Trait Anxiety Inventory (STAI) at T1 and T3 [23,24]. Either the psychologist (T1 to T3) or the recovery nurse (T4) will assess parental anxiety with the VAS.

Postoperative pain will be reported with 3 different instruments. The revised Faces Pain Scale (FPS-r) is a self-report measure designed for children to indicate pain intensity [25]. This measure will be used at T4 and T5. A recovery nurse, trained in administering the Face, Legs, Activity, Cry, and Consolability (FLACC) scale, will assess pain intensity at T4 [26,27]. This scale assesses nonverbal indicators of pain. The Parents’ Postoperative Pain Measure (PPPM) will be completed by parents at T5 [28].

Emergence delirium will be measured with the Pediatric Anesthesia Emergency Delirium (PAED) scale by the recovery nurse at T4 [29,30]. Finally, information regarding use of analgesics and healthcare use will be extracted from medical records.

Several factors associated with situational anxiety will be assessed because these may influence the efficacy of the VRE. Putative predictors are socioeconomic status, age, sex, type of surgery, preoperative child, and parental trait anxiety. Child trait anxiety will be assessed by parents with the Child Behavior Checklist (CBCL) at T1 [31]. Parental trait anxiety will be self-reported using the trait anxiety form (20 items) of the STAI.

### Statistical Analyses

To examine the effect of the intervention on the primary outcome, a repeated measures ANOVA will be conducted with child state anxiety level at T1 (baseline state anxiety) and T3 (anxiety during induction of anesthesia) as within variables and group (VRE versus CAU) as between variables.

For the secondary outcome measures, repeated measures ANOVA will be conducted with situational parental anxiety at T1 and T3 as within variables and group as between variables. Also, repeated measures ANOVAs will be performed using postoperative pain at T4 (in the recovery room) and T5 (at home) as outcomes, with group as between variables. The effect of the intervention on emergence delirium at T4 will be examined with an analysis of covariance (ANCOVA). Age and sex effects will be accounted for in all analysis.

Multiple linear regression analyses will be performed with change in child state anxiety between T1 and T3 and change in pain between T4 and T5 as outcomes. Predictor variables (ie, socioeconomic status, age, sex, type of surgery, preoperative child, and preoperative parental trait anxiety) will be included into the linear models to identify which factors influence VRE efficacy.

### Ethical Considerations

This study has been approved by the Medical Ethics Committee of the Erasmus Medical Center (MEC-2016-626). The study will be conducted according to the Helsinki Declaration.

## Results

The development of the VRE tool was finalized and participant recruitment began March 2017. The study to evaluate the efficacy of the VRE will be open for recruitment until September 2018. Data will be analyzed and scientific papers will be submitted for publication in the subsequent year.

## Discussion

### Principal Findings

There is a need to improve the psychological preparation of pediatric patients, as well as their parents, for surgery since elevated anxiety levels are highly prevalent. VRE has already been shown to be effective as a treatment for specific phobias in children. However, despite the fast-growing field of VR in medical care, the application of a VRE tool to reduce anxiety for surgical procedures in children has not been systematically studied. Since VR is a promising tool for improvement in health outcomes, high quality studies investigating innovative VR interventions are needed.

Here, we describe the development of a psychosocial VR intervention and the PREVIEW trial designed to test its efficacy. We expect the VRE to optimize the preparation of children for surgery under general anesthesia and diminish far-reaching maladaptive consequences, both psychologically and medically.

### Strengths and Limitations

In regular medical care, the explanations given to parents and children regarding surgery and anesthesia are mostly verbal of nature. VRE is primarily a visual, non-verbal intervention, so it can have a surplus value for young children, non-verbal children, and for children and parents who do not speak or fully comprehend their second language. Creating a less stressful experience for both children and their parents is in line with the emphasis on patient- and family-centered care [32]. Moreover, if VRE is proven to be effective, this easy to use tool can be implemented into standard medical care, engaging in secondary prevention. We would like to emphasize that, even with the use of modern technology, education provided by healthcare professionals of both pediatric patients and their parents is still absolutely necessary, especially for older children.

A limitation of our study is that it involves children undergoing elective day care surgery (more specifically, dental, oral, and ENT surgery). Therefore, the results of this study might not be generalizable to other types of surgeries.

### Conclusion

Preoperative anxiety in children is highly prevalent and there is a need to develop more effective strategies to reduce this anxiety. VRE is a promising tool to prepare pediatric patients for surgery in a child-friendly and efficacious way. We demonstrated that the development of a highly realistic virtual environment that replicates the operating theater is possible with the collaboration of a multidisciplinary team. We are now examining the efficacy of the VRE tool by means of an RCT. By focusing on preparing children for anesthesia and surgery with an innovative VRE tool, instead of distracting them, we hope to improve clinical and psychological outcomes.

## Abbreviations

3D: Three-Dimensional
ANOVA: Analysis of Variance
CAU: Care As Usual
CBCL: Child Behavior Checklist
ENT: Ear-Nose-Throat
FLACC: Face, Legs, Activity, Cry, and Consolability
FPS: Faces Pain Scale
mYPAS: modified Yale Preoperative Anxiety Scale
PAED: Pediatric Anesthesia Emergency Delirium
PPPM: Parents’ Postoperative Pain Measure
PREVIEW: PREoperative Virtual reality Intervention to Enhance Wellbeing
RCT: Randomized Controlled Trial
STAI: State-Trait Anxiety Inventory
STAIC: State-Trait Anxiety Inventory for Children
VAS: Visual Analogue Scale
VR: Virtual Reality
VRE: Virtual Reality Exposure
